# ZnO Doping-Induced Performance Boost in Co_2_TiO_4_/n-Si Schottky Self-Powered Photodetectors

**DOI:** 10.1021/acsomega.5c12653

**Published:** 2026-02-23

**Authors:** Ali Akbar HUSSAINI, Adem SARILMAZ, Faruk OZEL, Mehmet Okan ERDAL, Murat YILDIRIM

**Affiliations:** 1 Department of Biotechnology, 563761 Selcuk University, Konya 42130, Turkey; 2 Department of Metallurgical and Materials Engineering, 166263 Karamanoglu Mehmetbey University, Karaman 70200, Turkey; 3 Department of Mechanical Engineering, 175650 Recep Tayyip Erdogan University, Rize 53100, Turkey; 4 Meram Vocational School, 226846 Necmettin Erbakan University, Konya 42090, Turkey

## Abstract

In this study, we employed Co_2_TiO_4_ and synthesized ZnO-doped Co_2_TiO_4_ nanocomposites, which were utilized as interlayers in silicon-based Schottky photodetectors for optoelectronic characterization. Structural analyses using XRD, SEM, and EDX confirmed successful fabrication. The photodetectors were evaluated across a broad spectral range (351–1600 nm) and under varying light intensities. The incorporation of ZnO nanoparticles significantly enhanced the performance of the Co_2_TiO_4_-ZnO/n-Si device compared to its undoped counterpart. Notably, the responsivity (*R*) improved from 0.35 to 17.39 mA/W at 351 nm and from 1.75 to 23.76 mA/W at 1000 nm. Correspondingly, the specific detectivity increased by nearly an order of magnitude, surpassing 10^10^ Jones across much of the spectrum. The noise equivalent power (NEP) decreased drastically from 2.16 × 10^–10^ to 1.03 × 10^–11^ W·Hz^–1/2^ at 351 nm and from 4.33 × 10^–11^ to 7.53 × 10^–12^ W. Hz^–1/2^ at 1000 nm, indicating enhanced sensitivity. The external quantum efficiency (EQE) also improved from 0.13 to 6.43% at 351 nm, with sustained enhancement in the UV–vis-NIR regions. Under 20 mW/cm^2^ illumination, the Co_2_TiO_4_-ZnO/n-Si device exhibited a responsivity of 85.99 mA/W compared to 10.23 mA/W for the Co_2_TiO_4_/n-Si device, and a detectivity of 4.26 × 10^10^ Jones, significantly higher than 1.03 × 10^10^ Jones for the undoped counterpart. These results demonstrate that ZnO doping significantly improves light absorption, carrier transport, and signal-to-noise ratio, making the Co_2_TiO_4_-ZnO/n-Si photodetector a promising candidate for high-performance, broadband, self-powered photodetection applications.

## Graphical abstract

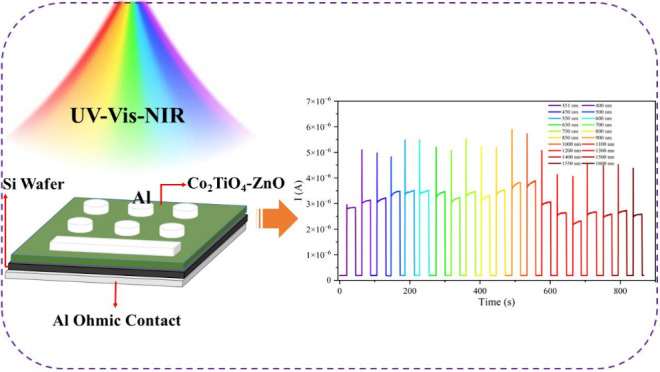

## 1 Introduction

Photodetectors (PDs), being crucial optoelectronic devices that convert light into electrical signals, have garnered significant interest due to their vast potential uses in flame detection,^1^ ozone monitoring,^2^ communication conversion,^3^ environmental assessment,^4^ imaging,^5^ night vision,^6^ materials identification,^7,8^ early tumor detection,^9^ health monitoring,^10^ astronomical research,^11^ and more. Traditional PDs require an energy source for the detection process, restricting their use. In contrast, self-powered PDs do not require this. Self-powered PDs featuring a straightforward device design are virtually maintenance-free, have minimal to no external power needs, operate wirelessly, are self-sufficient, and possess an extended lifespan. To put it differently, wireless, self-sustaining PDs are promising devices that can both improve the quality of life for people and encourage economic and technological advancement.^12,13^

Nanostructures exhibit unique optical, magnetic, and electrical properties owing to quantum effects, which are absent in their bulk counterparts.^14–17^ In semiconducting nanostructures, absorption of light with energy exceeding the bandgap promotes electrons from the valence band to the conduction band, generating free charge carriers. The method accomplishes the conversion of light into electronic signals, making semiconductor materials viewed as the most promising options for photodetectors.^18^ SiC, GaN, and some group II–V compound semiconductors typically have much greater thermal conductivity compared to Si, making them ideal for use in extreme conditions (such as elevated temperature and high power). The electron speed in these materials under high electric fields is typically greater than that in standard semiconductors, even though wide-bandgap semiconductors display comparatively lower mobilities for electrons and holes.^19^ Wide band gap semiconductors like SnO_2_, TiO_2_, and ZnO have garnered significant interest because of their potential uses in next-generation electronic and optoelectronic devices.^20–23^ Among these materials, zinc oxide thin films, which are n-type semiconductors, are particularly intriguing due to their impressive optical and electrical characteristics, as well as their strong chemical stability.^24^ Consequently, ZnO is extensively utilized in metal oxide semiconductor (MOS) gas sensors, transparent conducting oxide (TCO) films, light-emitting diodes (LEDs), transparent thin film transistors (TFTs), ultraviolet (UV) lasers, and particularly UV detectors.^25,26^ Specifically, the beneficial characteristics of a wide direct band gap (*E*_g_ = 3.37 eV), elevated exciton binding energy (60 meV), excellent radiation resistance, nontoxicity, strong sensitivity to the UV range, and straightforward, cost-effective production make the ZnO material system an optimal selection for developing high-performance UV photodetectors.^27,28^ While ZnO is widely used, its performance as a standalone material is often limited by high carrier recombination. In this study, we deviate from conventional single-component designs by synthesizing a Co_2_TiO_4_-ZnO nanocomposite to serve as the functional layer in an Al/Co_2_TiO_4_-ZnO/n-Si device structure. The innovation of this material system lies in the intrinsic heterojunctions formed at the nanoscale between the Co_2_TiO_4_ and ZnO phases. These internal interfaces facilitate efficient charge separation within the composite before the carriers are collected at the Si interface. Furthermore, the Co_2_TiO_4_ phase introduces sub-bandgap electronic states that modulate carrier transport, offering a distinct advantage in terms of sensitivity and photocurrent levels compared to previously reported binary oxide systems.^29^ This dual-interface approach—combining a bulk nanocomposite with a semiconductor substrate—provides a robust framework for self-powered photodetection.

In this study, we successfully synthesized novel Co_2_TiO_4_ and Co_2_TiO_4_-ZnO nanocomposites and characterized them using X-ray diffraction (XRD), scanning electron microscopy (SEM), and energy-dispersive X-ray spectroscopy (EDX). The synthesized nanocomposites were employed as interlayers in the fabrication of silicon-based Schottky photodetector devices. The performance of the devices was evaluated under varying light intensities across a broad spectral range encompassing the ultraviolet (UV), visible (VIS), and near-infrared (NIR) regions.

## 2 Experimental Details

### 2.1 Synthesis of Co_2_TiO_4_-ZnO Nanocomposite

The Co_2_TiO_4_-ZnO nanocomposite was prepared via colloidal synthesis route, similar to the procedure reported in our previous work.^30^ In this approach, commercial Co_2_TiO_4_ nanopowder was used as a solid component, while ZnO was integrated through in situ synthesis using zinc acetate. For this purpose, 183.48 mg (1 mmol) of Zn­(CH_3_CO_2_)_2_ and 81.38 mg of commercial Co_2_TiO_4_ were employed to obtain an equal weight ratio (1:1), considering that 1 mmol of zinc acetate corresponds to 81.38 mg of ZnO. The precursor materials were dispersed in 10 mL of oleylamine (OLA), which served as both solvent and coordinating agent. The reaction mixture was heated to 240 °C and maintained at this temperature for 5 min. During the synthesis, Co_2_TiO_4_ nanoparticles remained suspended in the reaction medium, allowing ZnO nucleation and growth to occur in the presence of Co_2_TiO_4_, leading to the formation of a nanocomposite structure rather than a simple physical mixture. After cooling the solution to 70 °C, the nanocomposite was collected by centrifugation using a toluene/ethanol mixture (3:1 v/v).

### 2.2 Fabrication of Schottky Photodetector Devices

Phosphorus-doped *n*-type silicon wafers (Wafer World, USA) were cut into 10 × 10 mm^2^ pieces and cleaned using the standard Radio Corporation of America (RCA) method.^31^ The substrates were subsequently treated with diluted hydrofluoric acid to remove residual surface oxides and contaminants. Aluminum was deposited on the backside of the wafers via physical vapor deposition (PVD), followed by annealing at 450 °C for 5 min in a nitrogen atmosphere. A 50 μL aliquot of Co_2_TiO_4_ or Co_2_TiO_4_-ZnO solution was drop-cast onto the front surface of each wafer and uniformly spread using a spin coater operated at 1500 rpm for 40 s. Metallic contacts were deposited on the nanocomposite-coated surface by PVD through a hole array mask, defining a contact area of 7.85 × 10^–3^ cm^2^. Figure 1a depicts a schematic illustration of the fabricated Co_2_TiO_4_/n-Si and Co_2_TiO_4_-ZnO/n-Si photodetector device, highlighting the Si wafer substrate, the Al ohmic back contact, and the top Al electrodes. The fabricated devices were characterized for their electrical and optoelectronic performance using a solar simulator (FY 7000) in combination with a Keithley 2400 source meter. Monochromatic illumination was provided by visible hard-coated band-pass filters (Thorlabs GmbH, Germany) with an average full width at half-maximum (fwhm) of 10 nm, spanning a broad spectral range from the ultraviolet (351 nm) through the visible and into the near-infrared region (up to 1600 nm).

**Figure 1:**
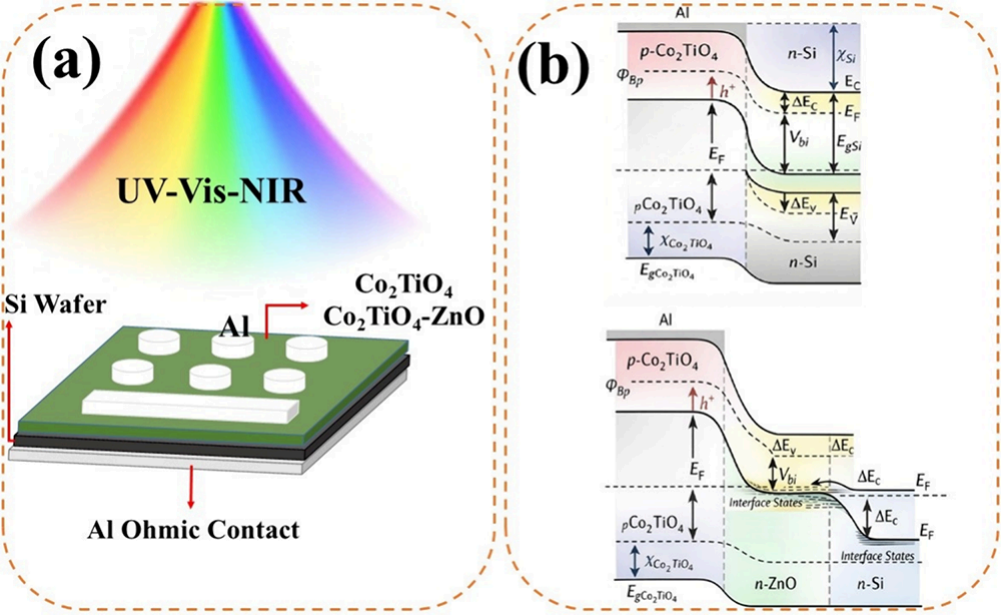
(a) Schematic illustration of the Co_2_TiO_4_/n-Si and Co_2_TiO_4_-ZnO/n-Si photodetector operating in the UV–Vis–NIR region. (b) Equilibrium energy band diagrams of the pristine Co_2_TiO_4_/n-Si and Co_2_TiO_4_-ZnO/n-Si heterojunctions, showing band alignment and carrier transport mechanisms.

Figure 1b shows the corresponding energy band diagrams of the heterojunctions under equilibrium conditions. When the Co_2_TiO_4_/n-Si heterojunction structure is analyzed, the base of the system consists of a phosphorus-doped n-type silicon (n-Si) wafer, while Co_2_TiO_4_ nanoparticles with a cubic spinel crystal structure function as the active layer. In this structure, the electronic properties of n-Si combine with the broad spectral absorption capability of Co_2_TiO_4_, enabling the device to operate over a wide wavelength range from the ultraviolet to the near-infrared (351–1600 nm). The Schottky barrier height, which is a key parameter of the energy band diagram formed at the interface of the two materials, has been experimentally determined to be approximately 0.76 eV for the pristine Co_2_TiO_4_/n-Si interface. The nanocomposite interface obtained by incorporating n-type wurtzite ZnO into the structure optimizes these barrier characteristics, accelerates charge carrier separation, and enables the system to operate with high sensitivity without the need for an external power source.

## 3 Results and Discussion

### 3.1 Structural and Morphological Characterization

X-ray diffraction (XRD) analysis was performed to confirm the crystalline nature of the synthesized Co_2_TiO_4_-ZnO composite and commercial Co_2_TiO_4_ nanopowder. Phase analysis of the obtained diffraction patterns (Figure 2a) was carried out using HighScore software, where phase identification was performed by comparison with the program’s reference database. According to these comparisons, commercial Co_2_TiO_4_ was determined to crystallize in a spinel structure, characterized by a cubic lattice arrangement (COD ID: 5910130). Additionally, it is seen that the composite comprises both spinel Co_2_TiO_4_ and wurtzite ZnO phases. The wurtzite ZnO phase crystallizes in a hexagonal lattice, corresponding to the *P*6_3_ *mc* space group (COD ID: 9004181). The diffraction peaks located at 30.1, 35.4, 43.1, 53.4, 56.8, and 62.5° were indexed to the (022), (131), (040), (242), (151), and (044) planes of the spinel Co_2_TiO_4_ phase, respectively. In addition, the peaks observed at 31.8, 34.4, 36.3, 47.4, 62.9, and 67.8° were assigned to the (100), (002), (101), (102), (103), and (112) planes of the wurtzite ZnO phase, respectively, in agreement with the standard diffraction data. The presence of these characteristic reflections, together with the absence of any additional impurity peaks in the diffraction patterns, confirms the phase purity of the synthesized materials.

**Figure 2:**
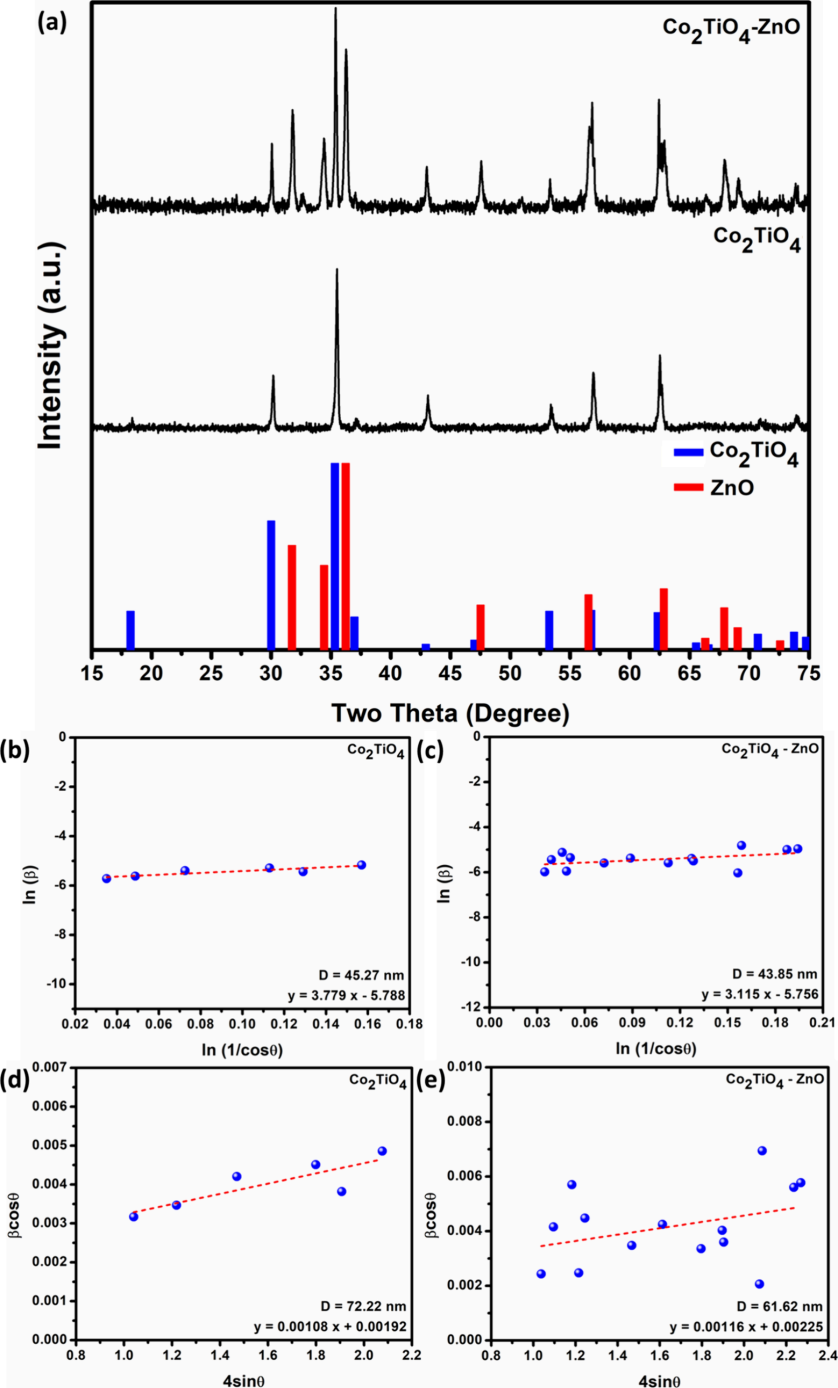
(a) XRD patterns of Co_2_TiO_4_ and Co_2_TiO_4_-ZnO are presented in comparison with standard diffraction patterns. ln­(β) vs ln­(1/cosθ) plots of (b) Co_2_TiO_4_ and (c) Co_2_TiO_4_-ZnO. Williamson–Hall graphs of (d) Co_2_TiO_4_ and (e) Co_2_TiO_4_-ZnO.

The crystallite sizes of Co_2_TiO_4_ and Co_2_TiO_4_-ZnO were evaluated through X-ray diffraction analysis using the modified Scherrer and the Williamson–Hall equations. In order to determine the crystallite size from these equations, the full width at half-maximum (fwhm) values of the diffraction peaks were required. The peak widths were determined by Voigt profile fitting, and the corresponding fwhm values were calculated using eq 1:^32^

$$\beta =0.5346xW_{L}+\sqrt{0.2166xW_{L}^{2}+W_{G}^{2}}$$ 1

where β is the fwhm calculated by using Gaussian (*W*_G_) and Lorentz (*W*_L_) peak broadening. The calculated fwhm values were employed in the modified Scherrer equation (eq 3), which is derived from the logarithmic form of the Scherrer equation (eq 2), and lnβ vs ln­(1/cosθ) graphs were plotted. From the linear fit of the plots shown in Figure 2b,c, the *y*-intercept corresponding to ln­(*K*λ/*D*) was determined.^33^ From this, the crystallite sizes were calculated to be 45.27 nm for Co_2_TiO_4_ and 43.85 nm for the Co_2_TiO_4_-ZnO composite.

$$\beta =\frac{K\lambda }{D}\cdot \frac{1}{\cos\theta }$$ 2

$$\ln\beta =\ln\;\frac{K\lambda }{D}+\ln\;\frac{1}{\cos\theta }$$ 3

where *K* is the shape factor, typically taken as 0.9, λ is the wavelength of the X-ray source (0.15405 nm for Cu K_α_), D represents the crystallite size, and θ is the diffraction angle.

The Williamson–Hall equation considers that the broadening of diffraction peaks arises from both the crystallite size and the lattice strain. Therefore, for a more accurate and precise determination, the Williamson–Hall equation given in eq 4 was employed. Williamson–Hall graphs (Figure 2d,e) were drawn based on eq 5 obtained by rearranging eq 4. By applying a linear fit to the Williamson–Hall plot, the *y*-intercept of the fitted line was identified.^33^ The crystallite sizes were calculated from the intercept to be 72.22 and 61.62 nm for Co_2_TiO_4_ and the Co_2_TiO_4_-ZnO composite, respectively. Moreover, the slope of the fitted line corresponds to microstrain, which was estimated as 1.08 × 10^–3^ for Co_2_TiO_4_ and 1.16 × 10^–3^ for Co_2_TiO_4_-ZnO composite.

$$\beta_{hkl}=\beta_{strain}+\beta_{crystal}=4\varepsilon \tan\theta +\left(\frac{K\lambda }{D}\cdot \frac{1}{\cos\theta }\right)$$ 4

$$\beta_{hkl}\cos\theta =4\varepsilon \sin\theta +\frac{K\lambda }{D}$$ 5

where ε represents microstrain.

The morphological and surface properties of the prepared electrodes for the photodetector were investigated using scanning electron microscopy (SEM). As shown in Figure 3a–d, Co_2_TiO_4_ and Co_2_TiO_4_-ZnO consist of agglomerated nanocrystals with irregular and quasi-spherical morphologies. The average particle size of Co_2_TiO_4_ nanopowder was calculated as 340 nm from Figure 3a,b. Additionally, Figure 3c,d shows that ZnO nanocrystals grew on the surface of Co_2_TiO_4_ particles during the synthesis of the nanocomposites, with an average particle size of 130 nm. The coating thicknesses of electrodes were measured from cross-sectional SEM images as ∼2.6 μm for Co_2_TiO_4_ and ∼3 μm for Co_2_TiO_4_-ZnO. Furthermore, elemental mapping images of Co_2_TiO_4_ and Co_2_TiO_4_-ZnO are given in Figure 4. These images show that all constituent elements are homogeneously distributed throughout the materials, and no impurity elements were detected.

**Figure 3:**
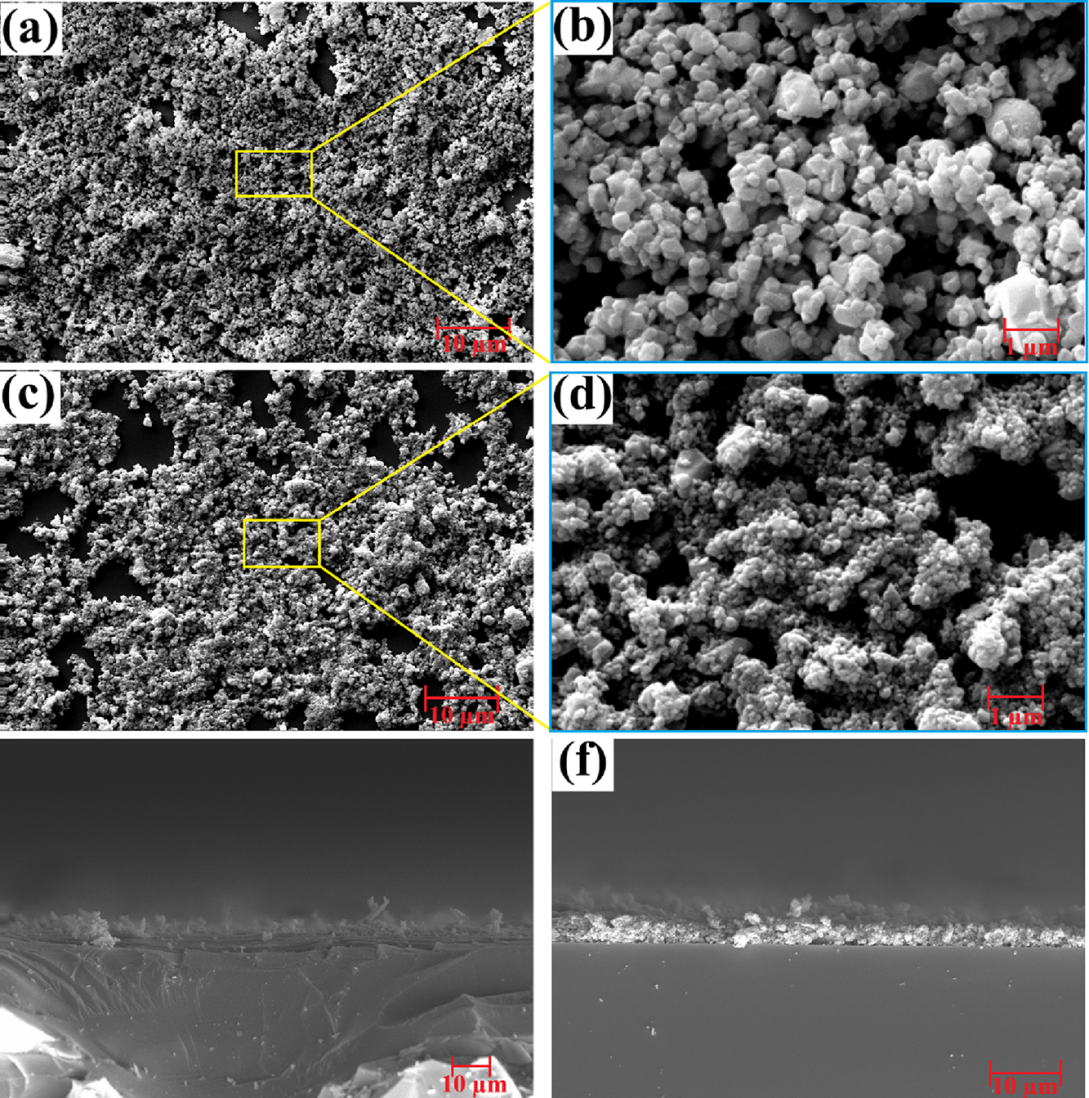
SEM images of the Co_2_TiO_4_ nanocomposite magnified at (a) 10,000×, and (b) 75,000×. SEM images of the Co_2_TiO_4_-ZnO nanocomposite magnified at (c) 10,000× and (d) 75,000×. Cross-section images of (e) Co_2_TiO_4_ and (f) Co_2_TiO_4_-ZnO thin film.

**Figure 4:**
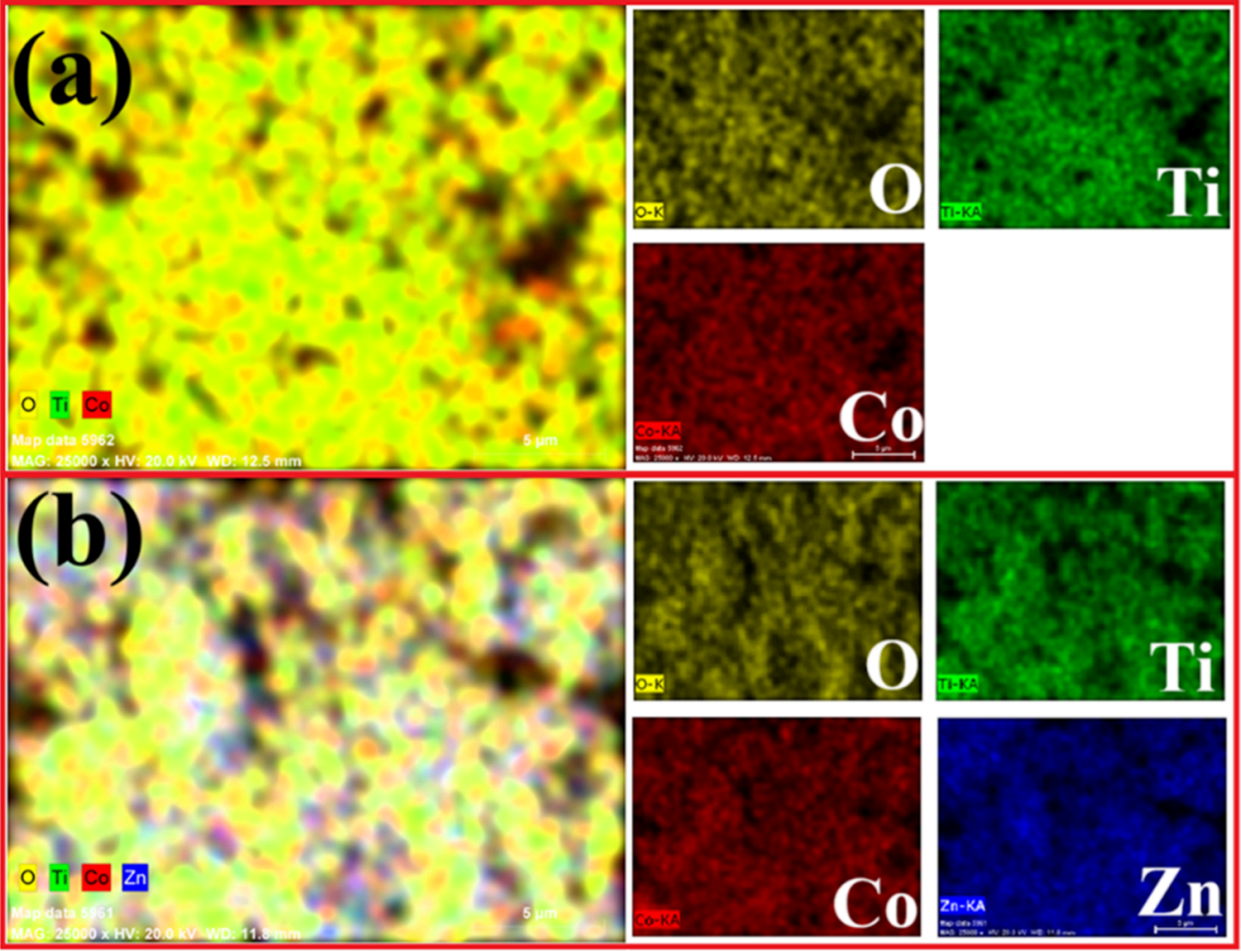
Elemental mapping images of (a) Co_2_TiO_4_ and (b) Co_2_TiO_4_-ZnO nanocomposites.

### 3.2 Electrical Properties

In the presence of light, carriers generated by photogenerated processes (electrons, holes) arise from both intrinsic and impurity absorption mechanisms, resulting in a change in the semiconductor’s conductivity. The carriers generated by light move under an applied bias and are gathered by the electrodes on either side, thus producing photocurrent in the output circuit.^34^

Figure 5 presents current–voltage (*I*–*V*) characteristics of Co_2_TiO_4_/n-Si and Co_2_TiO_4_-ZnO/n-Si photodetectors under various illumination intensities. Figure 5a and Figure 5b show the photocurrent response of Co_2_TiO_4_/n-Si and Co_2_TiO_4_-ZnO/n-Si, respectively, under increasing light intensities from 20 to 100 mW/cm^2^. In both devices, the current increases significantly with light intensity, confirming their photoconductive behavior. Notably, the Co_2_TiO_4_-ZnO/n-Si device exhibits higher photocurrent compared to the bare Co_2_TiO_4_/n-Si, indicating enhanced light absorption and charge carrier separation due to ZnO incorporation. Figure 5c compares both devices in the dark, where Co_2_TiO_4_-ZnO/n-Si shows slightly higher dark current, likely due to improved conductivity. Figure 5d further compares their performance under 100 mW/cm^2^ illumination, where the Co_2_TiO_4_-ZnO/n-Si device demonstrates a notably higher current response across the voltage range. Overall, the ZnO modification significantly improves the photodetection performance of the Co_2_TiO_4_-based device.

**Figure 5:**
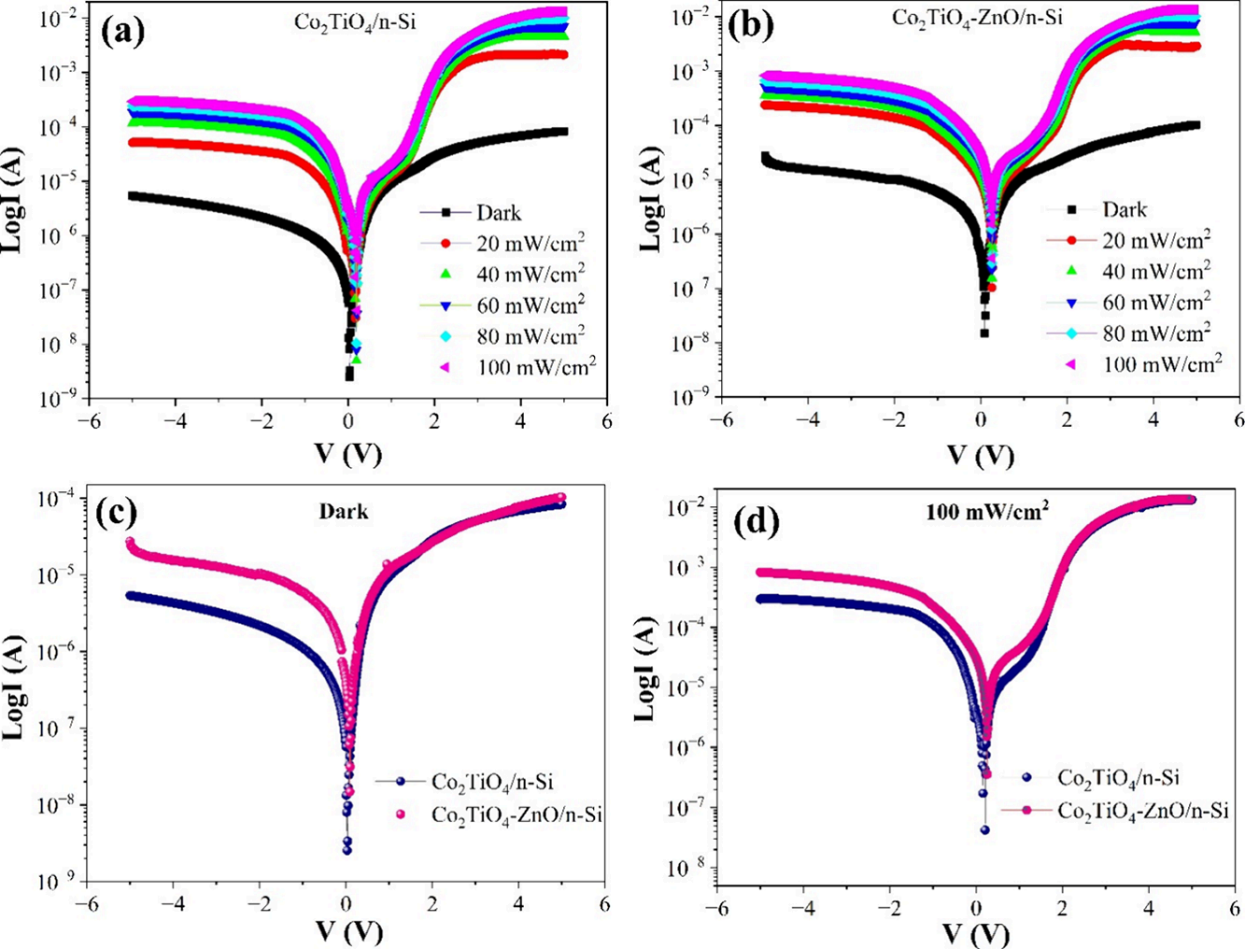
Semilogarithmic current–voltage (*I*–*V*) characteristics of (a) Co_2_TiO_4_/n-Si and (b) Co_2_TiO_4_-ZnO/n-Si devices under dark and different illumination intensities. Comparison of the two devices under (c) dark conditions and (d) illumination intensity of 100 mW/cm^2^.

The diode’s ideality factor, barrier height, and series resistance can be assessed through thermionic emission theory, alongside the Cheung and Norde analytical techniques. The ideality factor (*n*) is a fitting parameter used to modify the theoretical current–voltage relationship to align more closely with the slope found in experimental log­(*I*)–*V* data. Although an ideal diode has an ideality factor of *n* = 1, actual Schottky barrier diodes often show values exceeding 1.^35^ This variation is frequently ascribed to the effect of the applied bias on the actual barrier height.^36^ The barrier height (Φ_B_) indicates the potential difference between the metal’s Fermi level and the edge of the conduction or valence band of the semiconductor, depending on which charge carriers are predominant.^37^

Ideality factor and barrier height values were determined using the thermionic emission method. These values of Co_2_TiO_4_/n-Si and Co_2_TiO_4_-ZnO/n-Si devices were plotted against illumination and are shown in Figure 6a and Figure 6b, respectively. Ideality factors have increased and barrier height values decreased with increasing illumination. In an ideal rectifier, the ideality factor is equal to 1. Nonetheless, the effects of RS (series resistance), the presence of a nonuniform interface, the variations in barrier properties, and the thin oxide layer situated between the films could all lead to an ideality factor exceeding one.^38,39^ The rectifying ratio (RR) is an important parameter for evaluating the electrical performance of photodetectors. In this study, RR values were calculated at ±3 V for both Co_2_TiO_4_/n-Si and Co_2_TiO_4_-ZnO/n-Si devices, as shown in Figure 6c and Figure 6d, respectively. The Co_2_TiO_4_/n-Si device exhibited RR values of 15.69, 42.68, 31.64, 26.18, 24.57, and 24.74 at illumination intensities of 0, 20, 40, 60, 80, and 100 mW/cm^2^, respectively. In comparison, the Co_2_TiO_4_-ZnO/n-Si device showed RR values of 3.92, 13.88, 13.49, 11.65, 10.69, and 10.35 under the same illumination conditions. The lower RR values under dark conditions are attributed to the limited forward current. However, under illumination, a noticeable increase in forward current is observed, contributing to improved rectification behavior.

**Figure 6:**
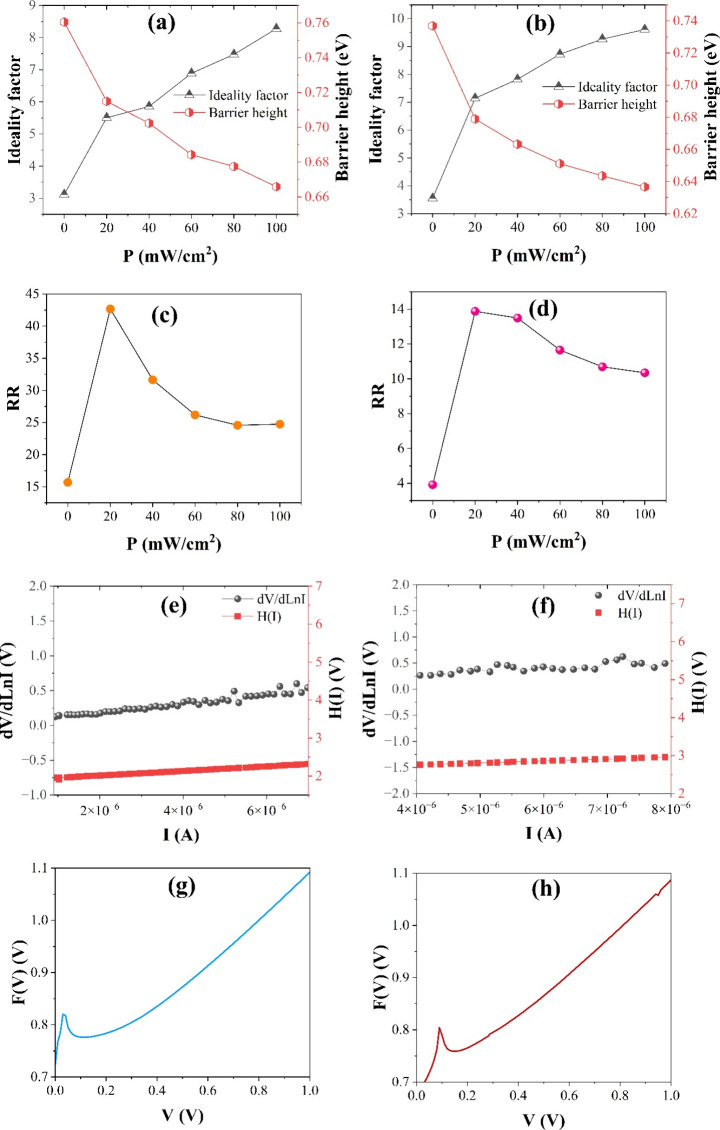
n-Φ_B_ plots of the (a) Co_2_TiO_4_/n-Si and (b) Co_2_TiO_4_-ZnO/n-Si devices. RR plots of the (c) Co_2_TiO_4_/n-Si and (d) Co_2_TiO_4_-ZnO/n-Si devices. Cheung plots of the (e) Co_2_TiO_4_/n-Si and (f) Co_2_TiO_4_-ZnO/n-Si devices. Norde plots of the (g) Co_2_TiO_4_/n-Si and (h) Co_2_TiO_4_-ZnO/n-Si devices.

To evaluate the electrical parameters of the Co_2_TiO_4_/n-Si and Co_2_TiO_4_-ZnO/n-Si devices, the Cheung method was applied to extract the ideality factor, barrier height, and series resistance. The resulting d*V*/d*l*n*I*–*I* and *H*(*I*)–*I* plots, obtained under dark conditions, are shown in Figure 6e and Figure 6f. According to this analysis, the Co_2_TiO_4_/n-Si device exhibited a barrier height of 0.743 eV and an ideality factor of 2.551, while the Co_2_TiO_4_-ZnO/n-Si device showed a slightly lower barrier height of 0.714 eV and a higher ideality factor of 3.556, as summarized in Table 1. The series resistance values, also derived from the Cheung plots, were 59.881 and 59.615 kΩ for the Co_2_TiO_4_/n-Si device, and 53.580 and 52.567 kΩ for the Co_2_TiO_4_-ZnO/n-Si device, indicating improved conductivity with ZnO incorporation. Additionally, the Norde method was used to cross-validate the barrier height and series resistance. Figure 6g,h displays the corresponding Norde plots. The barrier heights calculated via this method were slightly higher: 0.759 eV for Co_2_TiO_4_/n-Si and 0.749 eV for Co_2_TiO_4_-ZnO/n-Si. The series resistance values obtained from the Norde method were 82.500 kΩ for Co_2_TiO_4_/n-Si and 52.048 kΩ for Co_2_TiO_4_-ZnO/n-Si, further confirming the reduced resistance and enhanced charge transport in the ZnO-modified device.

**Table 1:** Diode Parameters of the Devices

Device	Saturation current (*I*_0_)	*n* (*I–V*)	*n* Cheung	**Φ**_b_ (*I–V*) (eV)	**Φ**_b_ Cheung (eV)	**Φ**_b_ Norde (eV)	*R*_s_ Cheung (kΩ (*H*(*I*)))	*R*_s_ Cheung ((kΩ (dln(*I*)))	*R*_s_ Norde (kΩ)
Co_2_TiO_4_/n-Si	1.46 × 10^–8^	3.117	2.551	0.760	0.743	0.759	59.881	59.615	82.500
Co_2_TiO_4_-ZnO/n-Si	3.63 × 10^–8^	3.549	3.556	0.737	0.714	0.749	53.580	52.567	52.048

### 3.3 Optoelectronic Properties

The time-dependent photocurrent response of a photodevice can be evaluated through current–time (*I*–*t*) measurements under intermittent illumination, either with an applied bias or in self-powered mode. In this study, the photocurrent response was assessed under self-powered conditions using varying solar light intensities. Figure 7a and Figure 7c display the *I*–*t* characteristics of Co_2_TiO_4_/n-Si and Co_2_TiO_4_-ZnO/n-Si heterojunction devices, respectively. Both devices exhibit a sharp and immediate increase in photocurrent upon illumination, indicating rapid photoresponse behavior. The photocurrent also increases progressively with rising light intensity, confirming the devices’ sensitivity to incident light power density. Figure 7b,d further highlights the rise and fall times, which are critical parameters in evaluating the response speed of photodetectors. The rise time corresponds to the duration required for the photocurrent to increase from 10 to 90% of its maximum value upon light exposure, while the fall time represents the time taken to decay from 90 to 10% upon cessation of illumination. The temporal response characteristics of the devices, including rise time and fall times, are essential indicators of their responsiveness and potential for high-speed photodetection. As shown in Figure 7b,d, the Co_2_TiO_4_/n-Si device exhibits a rise time of 0.55056 s and a fall time of 0.68026 s, indicating a relatively fast response and recovery upon light illumination and removal. In comparison, the Co_2_TiO_4_-ZnO/n-Si device shows a slightly longer rise time of 0.55555 s but a notably longer fall time of 1.12358 s.

**Figure 7:**
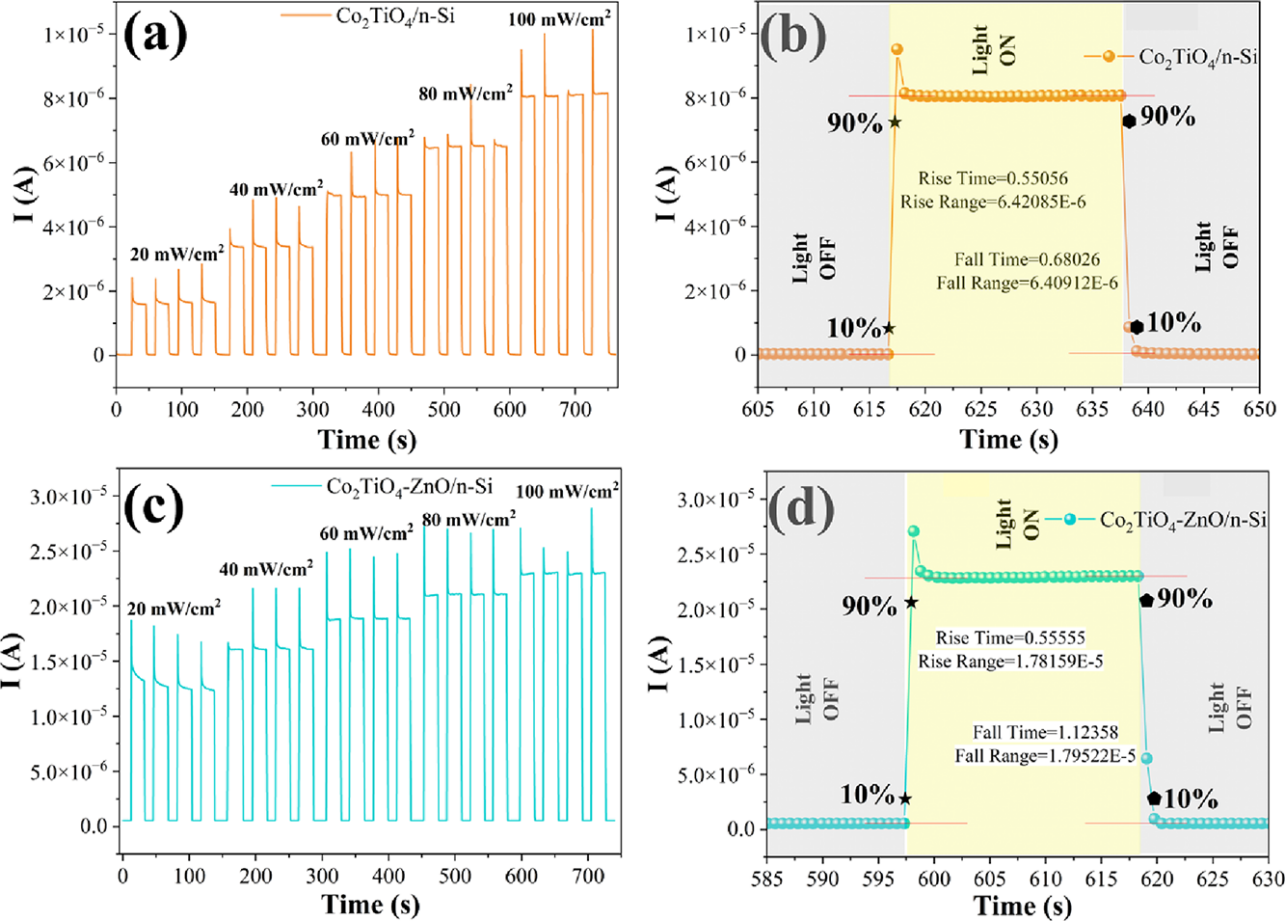
(a) *I*–*t* and (b) rise/fall time plot of the Co_2_TiO_4_/n-Si device. (c) *I*–*t* and (d) rise/fall time plot of the Co_2_TiO_4_-ZnO/n-Si device.

The rise time in both devices is nearly identical, suggesting that the addition of the ZnO layer does not significantly alter the charge generation speed under light excitation. However, the increased fall time in the ZnO-incorporated device could be attributed to enhanced charge trapping or prolonged carrier lifetime, which may be beneficial for charge storage applications but may slightly hinder rapid switching performance. The observed persistent photoconductivity (PPC) effect stems from a ″carrier lifetime″ extension caused by the spatial separation of photogenerated charges. In this process, holes trapped by surface states or deep-level defects (such as oxygen vacancies) are unable to undergo immediate recombination with conduction band electrons.^40–42^ These defects essentially create a kinetic barrier to recombination, prolonging the decay process. While this behavior enhances the photogain, it remains a limiting factor for applications requiring high-speed switching. Nevertheless, for practical sensing applications where high-frequency modulation is not the primary requirement, the Co_2_TiO_4_-ZnO/n-Si device provides an advantageous balance, offering substantially higher photocurrent levels without compromising the operational response speed required for real-world detection.

The key parameters that govern the performance of a photodetector include photocurrent (*I*_p_), photosensitivity (*K*), responsivity (*R*), noise equivalent power (NEP), and specific detectivity (D*). These characteristics are calculated using the following equations:^43–47^

$$I_{p}=I_{light}-I_{dark}$$ 6

$$K=\frac{I_{p}}{I_{dark}}$$ 7

$$R=\frac{I_{p}}{PA}$$ 8

$$\mathrm{NEP}=\frac{\sqrt{2eIdark}}{R}$$ 9

$$D^{*}=\frac{\sqrt{A}}{NEP}$$ 10

Here, *P* denotes the incident light power density and *A* represents the effective area of the photodetector. Responsivity (*R*) quantifies how effectively the device converts optical input into electrical output. Photosensitivity (*K*) reflects the ratio of the photocurrent to the dark current, indicating how sensitively the device responds to illumination. NEP represents the minimum optical power that can be detected above the noise level, serving as a critical metric for photodetector sensitivity. Specific detectivity (D*) expresses the device’s ability to detect weak optical signals and is normalized to the detector area and bandwidth. As illustrated in Figure 8a–d and summarized in Table 2, we calculated and plotted these performance parameters for each light intensity level to evaluate the overall photodetection capabilities of the devices.

**Figure 8:**
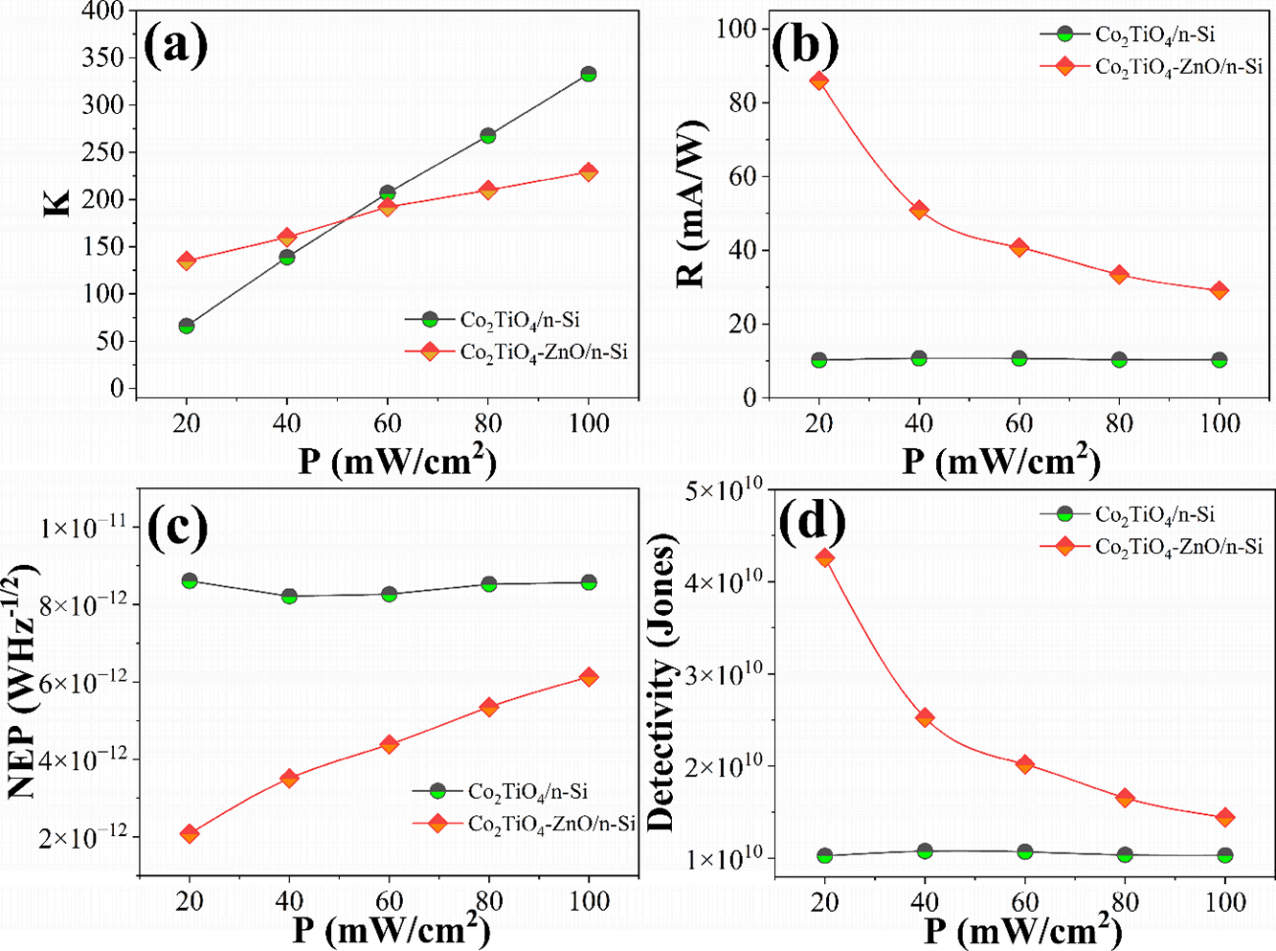
(a) Photosensitivity, (b) responsivity, and (c) NEP, and (d) detectivity plots of Co_2_TiO_4_/n-Si and Co_2_TiO_4_-ZnO/n-Si devices measured under solar illumination intensities.

**Table 2:** Photodetector Parameters under Various Solar Light Intensities

Device	Power (mW/cm^2^)	Photocurrent (A)	Photosensitivity -	Responsivity (mA/W)	Detectivity (Jones)	NEP (WHz^–1/2^)
Co_2_TiO_4_/n-Si	20	1.61 × 10^–6^	66.26	10.23	1.03 × 10^10^	8.61 × 10^–12^
	40	3.37 × 10^–6^	138.88	10.72	1.08 × 10^10^	8.22 × 10^–12^
	60	5.02 × 10^–6^	206.96	10.65	1.07 × 10^10^	8.27 × 10^–12^
	80	6.49 × 10^–6^	267.62	10.33	1.04 × 10^10^	8.53 × 10^–12^
	100	8.07 × 10^–6^	332.81	10.27	1.03 × 10^10^	8.57 × 10^–12^
Co_2_TiO_4_-ZnO/n-Si	20	1.35 × 10^–5^	135.00	85.99	4.26 × 10^10^	2.08 × 10^–12^
	40	1.60 × 10^–5^	160.00	50.96	2.52 × 10^10^	3.51 × 10^–12^
	60	1.92 × 10^–5^	192.00	40.76	2.02 × 10^10^	4.39 × 10^–12^
	80	2.10 × 10^–5^	210.00	33.44	1.66 × 10^10^	5.35 × 10^–12^
	100	2.29 × 10^–5^	229	29.17	1.45 × 10^10^	6.13 × 10^–12^

Figure 8a illustrates the photosensitivity plot in various light intensities. At all power levels, the Co_2_TiO_4_-ZnO/n-Si device consistently demonstrates a significantly higher photocurrent than the Co_2_TiO_4_/n-Si device. For instance, at 20 mW/cm^2^, Co_2_TiO_4_-ZnO/n-Si generates a photocurrent of 1.35 × 10^–5^ A, compared to 1.61 × 10^–6^ A for Co_2_TiO_4_/n-Si, which is approximately 8.4 times higher. This enhanced current output translates into improved photosensitivity, particularly noticeable at lower light intensities. However, the photosensitivity of both devices decreases with increasing power density, indicating some degree of saturation or recombination effects at higher illumination levels. Figure 8b shows the responsivity of the fabricated devices. At 20 mW/cm^2^, the responsivity of Co_2_TiO_4_-ZnO/n-Si reaches 85.99 mA/W, greatly surpassing the 10.23 mA/W observed in Co_2_TiO_4_/n-Si. Although the responsivity of the ZnO-modified device decreases at higher intensities, it remains superior across all tested conditions. NEP values of the devices in various illuminations is plotted in Figure 8c. NEP, the lowest detectable power per root bandwidth, is lower for the Co_2_TiO_4_-ZnO/n-Si device across all light intensities, indicating better noise performance. At 20 mW/cm^2^, the NEP is 2.08 × 10^–12^ W·Hz^–1/2^ compared to 8.61 × 10^–12^ W·Hz^–1/2^ for Co_2_TiO_4_/n-Si. Lower NEP values are desirable, and this metric further confirms the enhanced sensitivity and low-noise operation of the ZnO-incorporated device. Detectivity plots of the devices is shown in Figure 8d. Co_2_TiO_4_-ZnO/n-Si achieves a maximum detectivity of 4.26 × 10^10^ Jones at 20 mW/cm^2^, which is about four times higher than that of the Co_2_TiO_4_/n-Si device (1.03 × 10^10^ Jones). This highlights the superior capability of the ZnO-modified device to detect low-intensity signals with high precision. Although detectivity declines with increasing power in both devices, Co_2_TiO_4_-ZnO/n-Si maintains a consistently higher value.

Figure 9a and Figure 9b illustrate the wavelength-dependent photocurrent responses of the Co_2_TiO_4_/n-Si and Co_2_TiO_4_-ZnO/n-Si photodetectors, respectively, under zero-bias conditions. Illumination was achieved using a set of narrowband visible hard-coated band-pass filters (Thorlabs GmbH, Germany) with an average full width at half-maximum (fwhm) of 10 nm, covering a broad spectral range from the ultraviolet (UV) through the visible and into the near-infrared (NIR) region (351 to 1600 nm). This configuration enabled a comprehensive evaluation of the spectral response characteristics of each device. Both photodetectors exhibited broad spectral sensitivity, with measurable photocurrent responses across the entire tested range. However, the Co_2_TiO_4_-ZnO/n-Si device (Figure 9b) produced significantly higher photocurrent values compared to the Co_2_TiO_4_/n-Si device (Figure 9a) under identical illumination conditions. This enhancement is especially evident in the visible to NIR regions, indicating improved light absorption and charge separation due to the ZnO interlayer. The photocurrent trends generally peak within the visible range (around 600–800 nm) for both devices, followed by a gradual decline toward longer wavelengths, which is consistent with the photon energy distribution and the absorption characteristics of the materials involved. Importantly, all measurements were conducted under self-powered conditions (0 V applied bias), demonstrating the intrinsic photovoltaic behavior of the heterojunctions. This zero-bias operation underscores the suitability of these devices for low-power and energy-efficient photodetector applications across a broad spectral range.

**Figure 9:**
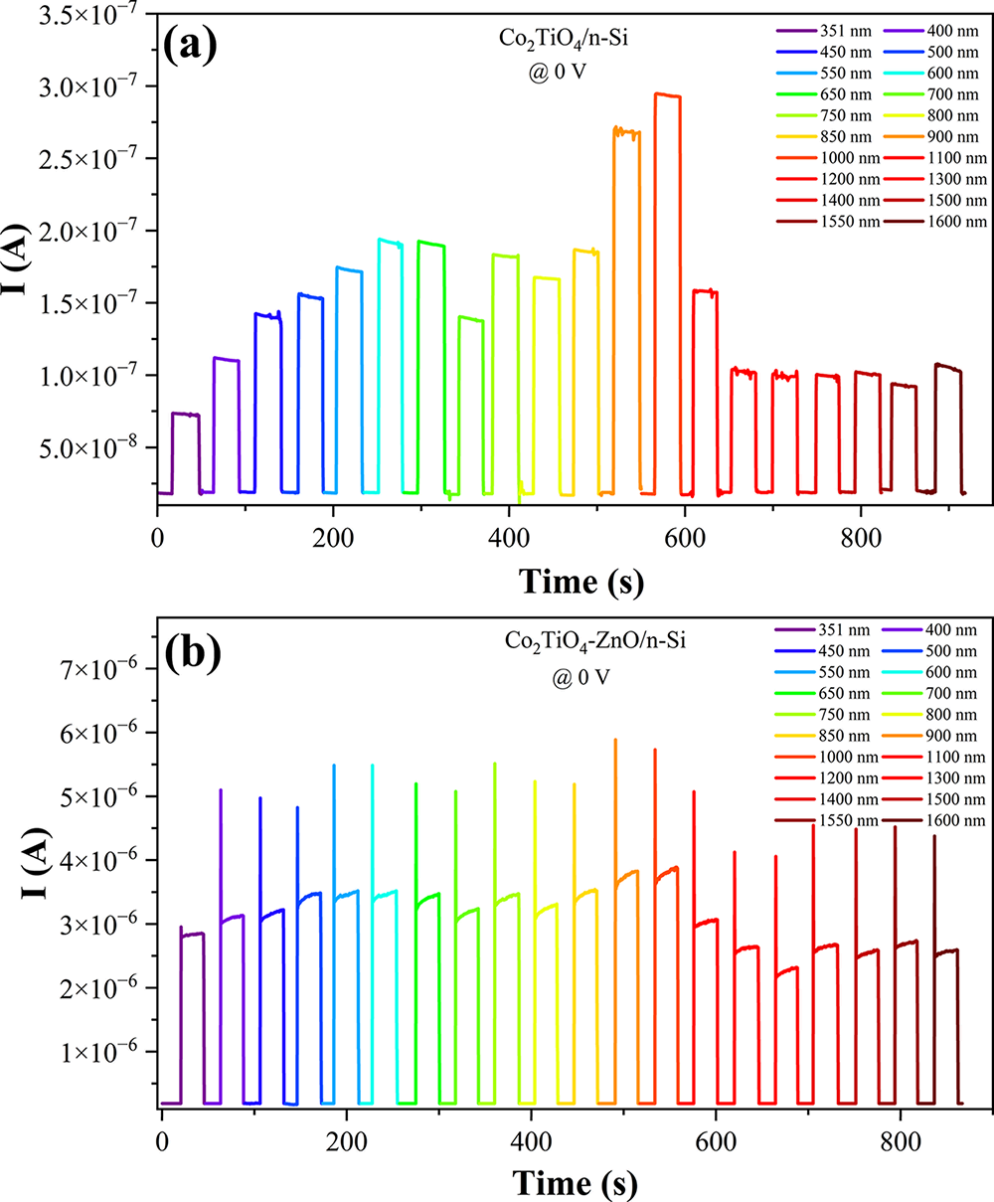
I-t plots of (a) Co_2_TiO_4_/n-Si and (b) Co_2_TiO_4_–ZnO/n-Si devices measured under various wavelengths.

Figure 10a–f and Table 3 summarize the spectral performance of Co_2_TiO_4_/n-Si and Co_2_TiO_4_-ZnO/n-Si photodetectors across a wavelength range of 351–1600 nm under self-powered operation (0 V bias). The data presented were derived from measured photocurrents using narrow band-pass filters and used to calculate key photodetection metrics.

**Figure 10:**
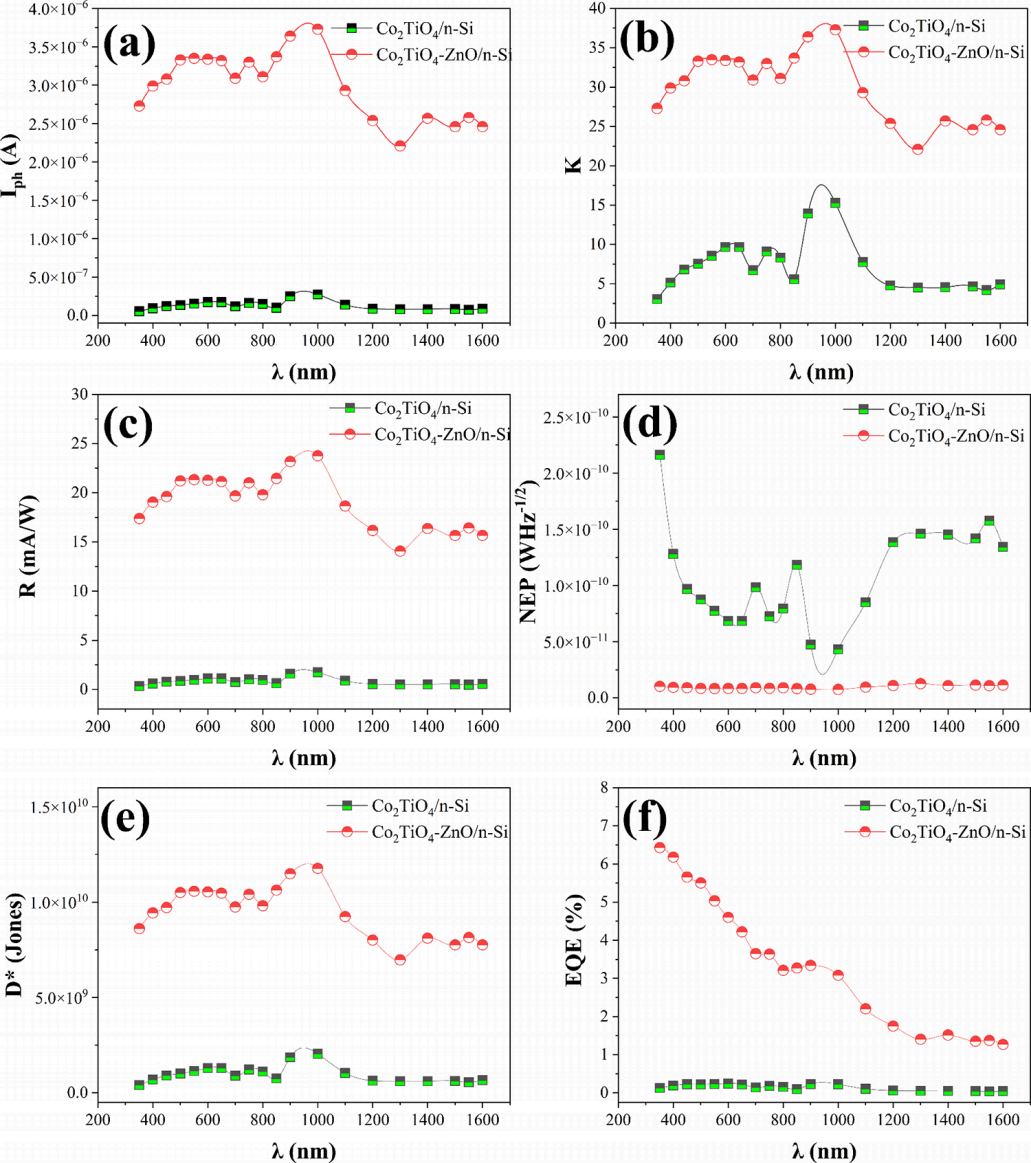
(a) *I*_ph_-λ, (b) *K*-λ, (c) *R*-λ, (d) NEP-λ, (e) D*-λ, and (f) EQE-λ plots of based devices measured over the UV–vis–NIR spectral range (351–1600 nm).

**Table 3:** Photodetector Parameters across UV–Vis-NIR Regions Obtained at Zero-Bias Voltage

	Co_2_TiO_4_/n-Si				Co_2_TiO_4_-ZnO/n-Si			
**λ (nm)**	**R**(mA/W)	D* (Jones)	**NEP** **(W Hz** ^**–1/2**^ **)**	**EQE (%)**	***R*** (mA/W)	D* (Jones)	**NEP** **(W Hz** ^**–1/2**^ **)**	**EQE (%)**
351	0.35	4.10 × 10^8^	2.16 × 10^–10^	0.13	17.39	8.61 × 10^9^	1.03 × 10^–11^	6.43
400	0.59	6.92 × 10^8^	1.28 × 10^–10^	0.19	19.04	9.44 × 10^9^	9.39 × 10^–12^	6.18
450	0.78	9.15 × 10^8^	9.69 × 10^–11^	0.23	19.62	9.72 × 10^9^	9.12 × 10^–12^	5.66
500	0.87	1.01 × 10^9^	8.76 × 10^–11^	0.22	21.21	1.05 × 10^10^	8.43 × 10^–12^	5.51
550	0.98	1.15 × 10^9^	7.74 × 10^–11^	0.23	21.34	1.06 × 10^10^	8.38 × 10^–12^	5.04
600	1.11	1.2 × 10^9^	6.85 × 10^–11^	0.24	21.27	1.05 × 10^10^	8.41 × 10^–12^	4.60
650	1.11	1.29 × 10^9^	6.85 × 10^–11^	0.22	21.15	1.05 × 10^10^	8.46 × 10^–12^	4.22
700	0.77	9.00 × 10^8^	9.85 × 10^–11^	0.14	19.68	9.75 × 10^9^	9.09 × 10^–12^	3.65
750	1.04	1.22 × 10^9^	7.27 × 10^–11^	0.18	21.02	1.04 × 10^10^	8.51 × 10^–12^	3.64
800	0.96	1.12 × 10^9^	7.94 × 10^–11^	0.16	19.81	9.81 × 10^9^	9.03 × 10^–12^	3.21
850	0.64	7.48 × 10^8^	1.18 × 10^–10^	0.10	21.46	1.06 × 10^10^	8.33 × 10^–12^	3.28
900	1.60	1.87 × 10^9^	4.75 × 10^–11^	0.23	23.18	1.15 × 10^10^	7.72 × 10^–12^	3.34
1000	1.75	2.05 × 10^9^	4.33 × 10^–11^	0.23	23.76	1.18 × 10^10^	7.53 × 10^–12^	3.08
1100	0.89	1.04 × 10^9^	8.51 × 10^–11^	0.11	18.66	9.25 × 10^9^	9.59 × 10^–12^	2.20
1200	0.55	6.40 × 10^8^	1.39 × 10^–10^	0.06	16.18	8.02 × 10^9^	1.11 × 10^–11^	1.75
1300	0.52	6.07 × 10^8^	1.46 × 10^–10^	0.05	14.08	6.97 × 10^9^	1.27 × 10^–11^	1.41
1400	0.52	6.10 × 10^8^	1.45 × 10^–10^	0.05	16.37	8.11 × 10^9^	1.09 × 10^–11^	1.52
1500	0.54	6.25 × 10^8^	1.42 × 10^–10^	0.05	15.67	7.76 × 10^9^	1.14 × 10^–11^	1.36
1550	0.48	5.62 × 10^8^	1.58 × 10^–10^	0.04	16.43	8.14 × 10^9^	1.09 × 10^–11^	1.38
1600	0.56	6.60 × 10^8^	1.34 × 10^–10^	0.05	15.67	7.76 × 10^9^	1.14 × 10^–11^	1.27

Figure 10 compares the photoresponse parameters of the Co_2_TiO_4_/n-Si and Co_2_TiO_4_-ZnO/n-Si photodetectors across the UV-NIR range. As shown in Figure 10a, the ZnO-modified device exhibits a consistently higher photocurrent over the entire spectrum, indicating more efficient photocarrier generation. This enhancement is further reflected in the photosensitivity (Figure 10b) and responsivity (Figure 10c), where the Co_2_TiO_4_-ZnO/n-Si device demonstrates markedly improved light-to-current conversion, particularly in the visible and near-infrared regions.

The improved responsivity directly translates into a higher specific detectivity (Figure 10e) and a substantially reduced noise equivalent power (Figure 10d), confirming the superior signal-to-noise characteristics of the ZnO-incorporated structure. In addition, the external quantum efficiency (Figure 10f) of the Co_2_TiO_4_-ZnO/n-Si device is significantly enhanced across the full wavelength range, with pronounced improvement in the UV–visible region, whereas the undoped device maintains comparatively low EQE values.

It is observed that the responsivity values recorded under broadband solar illumination (Table 2) are significantly higher than those obtained during monochromatic spectral measurements (Table 3). This disparity is attributed to the synergistic broad-spectrum absorption of the Co_2_TiO_4_-ZnO nanocomposite, where multiple energy states across the UV, visible, and NIR regions are excited simultaneously. Furthermore, high-intensity solar simulation triggers intensity-dependent photogating effects and trap-assisted gain.^48^ These mechanisms involve the filling of trap states at the Co_2_TiO_4_-ZnO and composite/Si interfaces, which significantly extends the carrier lifetime and increases internal gain under high photon flux. Such effects are considerably less pronounced under the lower-intensity monochromatic light used for spectral characterization, where the low density of photogenerated carriers is insufficient to saturate these trap states.

Overall, the incorporation of ZnO nanoparticles into the Co_2_TiO_4_ layer significantly improves broadband sensitivity, responsivity, detectivity, and noise performance. These results indicate that ZnO incorporation effectively enhances light absorption, carrier separation, and transport, making the Co_2_TiO_4_-ZnO/n-Si heterostructure a promising candidate for high-performance, broadband, self-powered photodetection.

Table 4 shows the comparison of the optoelectronic performance of the proposed Co_2_TiO_4_-ZnO photodetector with other reported ZnO-based interlayers, evaluating parameters such as responsivity, specific detectivity, and external quantum efficiency. The results demonstrate that Co_2_TiO_4_-ZnO-based device excels in self-powered operation (0 V), achieving a high responsivity of 23.76 mA/W and a detectivity of 1.18 × 10^10^ Jones at 1000 nm. While the ZnO:Nd device exhibits superior peak responsivity, it is important to note that the Co_2_TiO_4_-ZnO heterojunction provides a more versatile broadband response spanning from the UV (351 nm) to the near-infrared (1000 nm) spectrum without the need for an external power source. This performance significantly surpasses other self-powered alternatives, such as Tm-doped ZnO, particularly in the longer wavelength regions, confirming the effectiveness of the Co_2_TiO_4_-ZnO integration in enhancing charge separation and carrier collection efficiency.

**Table 4:** Comparison of Various Photodetectors

Interlayer	λ (nm)	*R* (mA/W)	D* (Jones)	EQE (%)	Applied bias (V)	Reference
Ni-doped ZnO	350	0.00752	7.92 × 10^8^		5	^49^
ZnO:Nd	385	730	1.18 × 10^11^	236		^50^
MoS_2_-ZnO	365	34.50	2.366 × 10^10^	11.696	5	^51^
	550	7.924	1.718 × 10^10^	2.686		
ZnAl	365	46.82	2.46 × 10^11^		0	^52^
Tm-doped ZnO	600	2.28	1.23 × 10^9^	4.72	0	^53^
	1000	3.72	2.26 × 10^9^	4.62		
Co_2_TiO_4_-ZnO	351	17.39	8.61 × 10^9^	6.43	0	This work
	550	21.34	1.06 × 10^10^	5.04		
	1000	23.76	1.18 × 10^10^	3.08		

## 4 Conclusions

The integration of ZnO nanoparticles into Co_2_TiO_4_ nanocomposites has been shown to drastically enhance the optoelectronic performance of silicon-based Schottky photodetectors. Across the full tested spectrum (351–1600 nm), the Co_2_TiO_4_-ZnO/n-Si device exhibited superior responsivity, detectivity, and quantum efficiency, while maintaining significantly lower NEP values. Key improvements include an increase in responsivity from 0.35 to 17.39 mA/W at 351 nm and from 1.75 to 23.76 mA/W at 1000 nm, a substantial enhancement in EQE (from 0.13 to 6.43% at 351 nm), and a reduction in NEP by nearly an order of magnitude. Under 20 mW/cm^2^ illumination, the device showed an 8.4× increase in photocurrent, a greater than 8× increase in responsivity, and a 4× improvement in detectivity compared to the undoped Co_2_TiO_4_ device. These results confirm that ZnO doping effectively boosts photocarrier generation, transport efficiency, and noise suppression, making Co_2_TiO_4_-ZnO/n-Si photodetectors strong candidates for next-generation, self-powered broadband light detection in UV, visible, and near-infrared applications.

## References

[1] Zhou C., Wang J., Shu L., Hu J., Xi Z., Li S., Tang W. (2025). ε-Ga2O3 Solar-Blind Photodetector: Pyroelectric
Effect and Flame Sensing Application. Vacuum.

[2] Xu M., Tian X., Lin Y., Xu Y., Tao J. (2024). Design and
Performance Evaluation of a Deep Ultraviolet LED-Based Ozone Sensor
for Semiconductor Industry Applications. Micromachines.

[3] Bao C., Yang J., Bai S., Xu W., Yan Z., Xu Q., Liu J., Zhang W., Gao F. (2018). High Performance and
Stable All-Inorganic Metal Halide Perovskite-Based Photodetectors
for Optical Communication Applications. Adv.
Mater..

[4] Li T., Hu G., Wu H., Ding L., Zhang J., Sun M., Li Y., Liu Z., Shao Y., Fang Y., Qiao Y., Shen L., Lin Y. (2024). Highly Sensitive Water Pollution
Monitoring Using Colloid-Processed Organic Photodetectors. Nat. Water.

[5] Chen H., Xi N., Song B., Chen L., Zhao J., Lai K. W. C., Yang R. (2013). Infrared Camera Using
a Single Nano-Photodetector. IEEE Sens. J..

[6] Rutz, F. ; Bächle, A. ; Aidam, R. ; Niemasz, J. ; Bronner, W. ; Zibold, A. ; Rehm, R. InGaAs SWIR Photodetectors for Night Vision. In Infrared Technology and Applications XLV; Fulop, G. F. ; Hanson, C. M. ; Andresen, B. F. , Eds.; SPIE, 2019; p 37. 10.1117/12.2518634.

[7] Manakkakudy
Kumaran A., De Iacovo A., Ballabio A., Frigerio J., Isella G., Colace L. (2024). Waste Material Classification Based
on a Wavelength-Sensitive Ge-on-Si Photodetector. Sensors.

[8] Hussaini A.
A., Yılmaz K., Karaman M., Yıldırım M. (2025). Self-Powered
Polyaniline/Si NIR Photodetectors for Waste Classification: Fabrication,
Optimization, and Application. ACS Appl. Electron.
Mater..

[9] Xie H., Pan Q., Wu D., Qin F., Chen S., Sun W., Yang X., Chen S., Wu T., Chi J., Huang Z., Wang H., Zhang Z., Chen B., Carmeliet J., Su M., Song Y. (2022). Lateral Heterostructured
Vis–NIR Photodetectors with Multimodal Detection for Rapid
and Precise Classification of Glioma. ACS Nano.

[10] Wu Z., Zhai Y., Kim H., Azoulay J. D., Ng T. N. (2018). Emerging
Design and Characterization Guidelines for Polymer-Based Infrared
Photodetectors. Acc. Chem. Res..

[11] Rieke G. H. (2007). Infrared
Detector Arrays for Astronomy. Annu. Rev. Astron.
Astrophys..

[12] Lee G. J., Choi C., Kim D., Song Y. M. (2018). Bioinspired Artificial
Eyes: Optic Components, Digital Cameras, and Visual Prostheses. Adv. Funct. Mater..

[13] Hu X., Li X., Li G., Ji T., Ai F., Wu J., Ha E., Hu J. (2021). Recent Progress of Methods to Enhance Photovoltaic
Effect for Self-Powered Heterojunction Photodetectors and Their Applications
in Inorganic Low-Dimensional Structures. Adv.
Funct. Mater..

[14] Nene A., Antarnusa G., Dulta K., Sadeghzade S., Wang L., Ravikumar C. H., Aman J., Khongthaw B., Kandwal A., Somani P., Kumar A., Ramachandran K., Subramaniam V., Galluzzi M., Dou S., Liu X. (2025). Au–Ag
Bimetallic Nanoparticles: Synthesis, Structure, and Application in
Sensing. ChemPhysMater..

[15] Ma H., Chen Y., Li H., Fu Y., Sun D., Wang G., Guo X., Dou S., Subramaniam V., Kumar A., Ramachandran K., Liu X. (2025). Fabricating Α-MnO
2 @NiMoO 4 Heterostructure Architecture With Superior Photoelectrocatalytic
Water Purification. EcoEnergy.

[16] Li N., Shang Z., Guo E., Lu Q., Wei M., Ji X.-Y., Liu X. (2025). Identifying Dynamic
Active Sites
in Ni Co1-MoO4 Nanotubes for Enhanced Electrocatalytic Hydrogen Evolution
Reaction. Chin. Chem. Lett..

[17] Ma H., Chen Y., Jin H., Wang X., Wang G., Fu Y., Wang P., Subramaniam V., Ramachandran K., Liu X. (2025). Constructing String-Cage Structure of α-MnO2@CoS2 Photoelectrocatalyst
for Efficient Detoxification Sulfonamides Wastewater. Sci. Energy Environ..

[18] Teng F., Hu K., Ouyang W., Fang X. (2018). Photoelectric Detectors Based on
Inorganic P-Type Semiconductor Materials. Adv.
Mater..

[19] Xie C., Lu X., Tong X., Zhang Z., Liang F., Liang L., Luo L., Wu Y. (2019). Recent Progress in Solar-Blind Deep-Ultraviolet Photodetectors
Based on Inorganic Ultrawide Bandgap Semiconductors. Adv. Funct. Mater..

[20] Shewale P. S., Patil S. I., Uplane M. D. (2010). Preparation of Fluorine-Doped Tin
Oxide Films at Low Substrate Temperature by an Advanced Spray Pyrolysis
Technique, and Their Characterization. Semicond.
Sci. Technol..

[21] Kumar M., Saravanan A., Joshi S. A., Chen S.-C., Huang B.-R., Sun H. (2024). High-Performance
Self-Powered UV Photodetectors Using SnO2 Thin Film
by Reactive Magnetron Sputtering. Sensors Actuators
A Phys..

[22] Kazmi J., Abbas A., James Young D., Hussain Shah J., Ahmad W., Shoaib Ahmad Shah S., Raza Ali Raza S., Ambri Mohamed M., Govorov A. O., Wang Z. (2025). ZnO Nanowire UV Photodetectors:
At the Intersection of Flexibility, Biocompatibility, and Visible
Blindness. Mater. Today.

[23] Shewale P. S., Lee N. K., Lee S. H., Kang K. Y., Yu Y. S. (2015). Ti Doped
ZnO Thin Film Based UV Photodetector: Fabrication and Characterization. J. Alloys Compd..

[24] Shewale P. S., Agawane G. L., Shin S. W., Moholkar A. V., Lee J. Y., Kim J. H., Uplane M. D. (2013). Thickness Dependent
H2S Sensing Properties
of Nanocrystalline ZnO Thin Films Derived by Advanced Spray Pyrolysis. Sensors Actuators B Chem..

[25] Ortel M., Wagner V. (2013). Leidenfrost Temperature
Related CVD-like Growth Mechanism
in ZnO-TFTs Deposited by Pulsed Spray Pyrolysis. J. Cryst. Growth.

[26] Xu Q. A., Zhang J. W., Ju K. R., Yang X. D., Hou X. (2006). ZnO Thin Film
Photoconductive Ultraviolet Detector with Fast Photoresponse. J. Cryst. Growth.

[27] He Y., Zhang W., Zhang S., Kang X., Peng W., Xu Y. (2012). Study of the Photoconductive
ZnO UV Detector Based on the Electrically
Floated Nanowire Array. Sensors Actuators A
Phys..

[28] Jandow N. N., Yam F. K., Thahab S. M., Abu Hassan H., Ibrahim K. (2010). Characteristics of ZnO MSM UV Photodetector with Ni
Contact Electrodes on Poly Propylene Carbonate (PPC) Plastic Substrate. Curr. Appl. Phys..

[29] Venegas C. J., Gutierrez F. A., Eguílaz M., Marco J. F., Reeves-McLaren N., Rivas G. A., Ruiz-León D., Bollo S. (2019). Co2TiO4/Reduced Graphene
Oxide Nanohybrids for Electrochemical Sensing Applications. Nanomaterials.

[30] Kuru T., Sarilmaz A., Aslan E., Ozel F., Hatay Patir I. (2025). Rational Design
of ZnO/SrTiO3 S-Scheme Heterojunction for Photo-Enhanced Piezocatalytic
Hydrogen Production. Appl. Surf. Sci..

[31] Metin Ö., Aydoǧan Ş., Meral K. (2014). A New Route for the
Synthesis of Graphene Oxide-Fe3O4 (GO-Fe3O4) Nanocomposites and Their
Schottky Diode Applications. J. Alloys Compd..

[32] Solanki R. G., Rajaram P., Bajpai P. K. (2018). Growth,
Characterization and Estimation
of Lattice Strain and Size in CdS Nanoparticles: X-Ray Peak Profile
Analysis. Indian J. Phys..

[33] Nazari N., Golzan M. M., Mabhouti K. (2024). Study of Urbach
Energy and Kramers–Kronig
on Mn and Zn Doped NiFe2O4 Ferrite Nanopowder for the Determination
of Structural and Optical Characteristics. Sci.
Rep..

[34] Shi L., Chen K., Zhai A., Li G., Fan M., Hao Y., Zhu F., Zhang H., Cui Y. (2021). Status and Outlook
of Metal–Inorganic Semiconductor–Metal Photodetectors. Laser Photon. Rev..

[35] Çolpan M. H. (2025). Investigation
Device Parametersof BPhen­(C24H16N2) Thin Film Based Photodiode. Int. J. Adv. Nat. Sci. Eng. Res..

[36] Nicholls J. R. (2021). Electron
Trapping Effects in SiC Schottky Diodes: Review and Comment. Microelectron. Reliab..

[37] Gaspari, F. 2.10 Semiconductors. In Comprehensive Energy Systems; Elsevier, 2018; pp 266–302. 10.1016/B978-0-12-809597-3.00221-2.

[38] Kacus H., Yilmaz M., Incekara U., Kocyigit A., Aydogan S. (2021). The Photosensitive
Activity of Organic/Inorganic Hybrid Devices Based on Aniline Blue
Dye: Au Nanoparticles (AB@Au NPs). Sensors Actuators
A Phys..

[39] Karataş Ş., Berk N. (2022). Performance of the Illumination Dependent Electrical
and Photodiode Characteristic of the Al/(GO:PTCDA)/p-Si Structures. Opt. Mater. (Amst)..

[40] Moore J., Thompson C. (2013). A Phenomenological Model for the
Photocurrent Transient
Relaxation Observed in ZnO-Based Photodetector Devices. Sensors.

[41] Ton-That C., Lem L. L. C., Phillips M. R., Reisdorffer F., Mevellec J., Nguyen T.-P., Nenstiel C., Hoffmann A. (2014). Shallow Carrier
Traps in Hydrothermal ZnO Crystals. New J. Phys..

[42] Liu K., Sakurai M., Aono M. (2010). ZnO-Based Ultraviolet Photodetectors. Sensors.

[43] Yıldırım F., Taşkın M., Ahmed A. B., Benhaliliba M., İncekara Ü., Aydoǧan Ş. (2025). Enhanced
Photoresponse in MIS-Type UV-Vis Photodetector via Biocompatible Glutathione­(C10H17N3O6S)
Interlayer: Theoretical and Experimental Analysis. Emergent Mater..

[44] Yilmaz M., Yaman M., Yildirim F., Nuhzat S., Aydogan S. (2025). A Self-Powered
and Cost-Effective RGO/n-Si Photodetector with Broad Spectral Response
Including Visible, UV, and near-IR Regions. Mater. Sci. Eng., B.

[45] Yildirim F., Yaman M., Yilmaz M., Koçyiğit A., Aydoğan Ş. (2025). Self-Powered High-Performance Broadband
Heterojunction Photodetector Based on Nitrogen- and Boron-Doped Graphene
Oxide. ACS Appl. Electron. Mater..

[46] Çolpan M. H. (2025). Performance
Analysis of Graphene Oxide Based Photodetector for Broad Spectrum
Light Sensing. Gazi Univ. J. Sci. Part A Eng.
Innov..

[47] Çolpan M. H. (2025). UV-Vis-NIR
Photodetector Produced Using Lavandula Angustifolia Extract: A Green
Approach. Int. J. Adv. Nat. Sci. Eng. Res..

[48] Konstantatos G., Sargent E. H. (2010). Nanostructured
Materials for Photon Detection. Nat. Nanotechnol..

[49] Abbasi F., Zahedi F., Yousefi M. h. (2021). Fabricating and Investigating High
Photoresponse UV Photodetector Based on Ni-Doped ZnO Nanostructures. Opt. Commun..

[50] Poul
Raj I. L., Valanarasu S., Hariprasad K., Ponraj J. S., Chidhambaram N., Ganesh V., Ali H. E., Khairy Y. (2020). Enhancement of Optoelectronic Parameters of Nd-Doped
ZnO Nanowires for Photodetector Applications. Opt. Mater. (Amst)..

[51] Gautam C., Verma A., Chaudhary P., Yadav B. C. (2022). Development of 2D
Based ZnO–MoS2 Nanocomposite for Photodetector with Light-Induced
Current Study. Opt. Mater. (Amst)..

[52] Sun S., Li W., Zhang Y., Gao Q., Zhang N., Qin Y., Feng W. (2025). Mixed Metal Oxide Heterojunction
for High-Performance Self-Powered
Ultraviolet Photodetection. Small.

[53] Karaca A., Yıldız D. E., Hussaini A. A., Unal F., Yıldırım M. (2025). Optoelectrical
Characterization of
a UV–Vis–NIR Broadband Photodetector Based on Tm-Doped
ZnO. Phys. B Condens. Matter.

